# Mitochondrial Dysfunction in the Cardio-Renal Axis

**DOI:** 10.3390/ijms24098209

**Published:** 2023-05-03

**Authors:** Nerea Mendez-Barbero, Jorge Oller, Ana B. Sanz, Adrian M. Ramos, Alberto Ortiz, Marta Ruiz-Ortega, Sandra Rayego-Mateos

**Affiliations:** 1 Laboratory of Vascular Pathology, IIS-Fundación Jiménez Díaz, 28040 Madrid, Spain; nerea.mendez@quironsalud.es (N.M.-B.); jorge.oller@quironsalud.es (J.O.); 2 Centro de Investigación Biomédica en Red de Enfermedades Cardiovasculares (CIBERCV), Faculty of Medicine and Biomedicine, Universidad Alfonso X El Sabio, 28037 Madrid, Spain; 3 Spain Nephrology Laboratory, IIS-Fundación Jiménez Díaz-Universidad Autónoma, 28040 Madrid, Spain; asanz@fjd.es (A.B.S.); amramos@fjd.es (A.M.R.); aortiz@fjd.es (A.O.); 4 REDINREN Spain/Ricors2040, 28029 Madrid, Spain; 5 Cellular Biology in Renal Diseases Laboratory, IIS-Fundación Jiménez Díaz-Universidad Autónoma, 28040 Madrid, Spain; mruizo@fjd.es

**Keywords:** mitochondrial dysfunction, kidney disease, cardiovascular disease, oxidative stress, treatment

## Abstract

Cardiovascular disease (CVD) frequently complicates chronic kidney disease (CKD). The risk of all-cause mortality increases from 20% to 500% in patients who suffer both conditions; this is referred to as the so-called cardio-renal syndrome (CRS). Preclinical studies have described the key role of mitochondrial dysfunction in cardiovascular and renal diseases, suggesting that maintaining mitochondrial homeostasis is a promising therapeutic strategy for CRS. In this review, we explore the malfunction of mitochondrial homeostasis (mitochondrial biogenesis, dynamics, oxidative stress, and mitophagy) and how it contributes to the development and progression of the main vascular pathologies that could be affected by kidney injury and vice versa, and how this knowledge may guide the development of novel therapeutic strategies in CRS.

## 1. Introduction

The incidence of heart failure and chronic kidney disease (CKD) is increasing worldwide [1]. The cardio-renal syndrome (CRS) was described in 2010 by the Acute Dialysis Quality Initiative (ADQI) as a heart and kidney disorder that is produced by an acute or chronic dysfunction in any of these organs that may induce a dysfunction of the other [2,3]. The global prevalence of CKD is estimated at 850 million people, of whom 10–47% had cardiovascular disease (CVD) [4,5]. CVD is the main cause of death in the CKD population [4]. In patients with heart failure, kidney failure increased the risk of all-cause mortality from 20% to 500% [6,7]. Indeed, CRS is identified as a public health burden due to its poorly understood pathogenesis and suboptimal treatment possibilities [8]. Currently, there are five types of CRS: (1) type-I CRS (acute cardiac failure that induces acute renal failure); (2) type-II CRS (chronic cardiac failure that leads chronic renal failure); (3) type-III CRS (acute kidney injury aggravating heart failure); (4) type-IV CRS (chronic kidney failure aggravating heart failure); and (5) type-V CRS (concurrent chronic cardiac and renal failure) [9]. However, due to the current controversy around these classifications, an updated CRS classification needs to be established.

Mitochondrial dysfunction is thought to play a key role in cardiovascular and kidney disease due to the high content of mitochondria and oxygen consumption in these tissues [10,11]. Mitochondria rapidly respond to insults and try to maintain homeostasis through changes in morphology and bioenergetics and upregulating self-renewal/degradation, and could be a bridge player, connecting the kidneys and heart in CRS [12,13]. Altered mitochondrial homeostasis that begins in the kidney as well as in the cardiovascular system may contribute to impaired cardiovascular and renal function independently or both at the same time through metabolism changes, chronic inflammation, oxidative stress, calcium dynamics, fibrosis, and autophagy [14,15,16].

Mitochondrial homeostasis changes could be not only a consequence of cardio-renal diseases, but also a possible common denominator between the kidney and cardiovascular system in the development of CRS. However, the vast majority of the studies about this field are focused on one pathology or another, but not how one affects the other (CRS). Currently, there are pathological elements targeting mitochondria that can commonly affect both renal and cardiovascular tissues simultaneously, such as circulating uremic toxins or hypertension. Studies show how the presence of uremic toxins resulting from renal malfunction can induce mitochondrial damage at the cardiovascular level [17]. On the other hand, hypertension is a common pathological event in CVD and CKD patients. Renal mitochondrial oxidative stress has been associated with renin–angiotensin system activation [18], leading to increased Angiotensin II and the subsequent induction of oxidative stress and, therefore, mitochondrial dysfunction in cardiovascular tissues, participating in the progression of damage [19]. These common pathological events demonstrate the close relationship between CKD–CVD and the important role of mitochondria in the context of CRS.

In this article, we summarize the role of mitochondrial homeostasis dysfunction in processes such as biogenesis, dynamics, oxidative stress, and mitophagy in the main vascular pathologies that could be affected by kidney injury and vice versa, and discuss novel therapeutic strategies for CRS. At the moment, more studies focused on the communication of the cardiovascular and kidney organ are needed to elucidate its crosstalk during pathological situations.

## 2. Mitochondrial Function under Physiological Conditions

Mitochondria are essential organelles whose main function is ATP production. However, lot of works have pointed out the functions of these organelles in processes such as cell death, intracellular calcium signaling, inflammatory response, metal ion homeostasis, redox state maintenance, phospholipid trafficking, and biosynthetic pathways. As such, the number and functionality of mitochondria is key to cell integrity and viability. In response to cell demands, mitochondria can change their morphology and number through mitochondrial biogenesis to generate new mitochondria and through mitochondrial dynamics involving fusion and fission. In mitochondrial fusion, two neighboring mitochondria join. This helps to keep them healthy and increases the capacity for oxidative phosphorylation during energy starvation [20,21]. Mitochondrial fission helps to remove damaged mitochondria by mitophagy and is also necessary for redistributing mitochondrial contents to daughter cells during mitosis [21].

Heart cells together with vessel wall cells conform to a high metabolic consumption and one of the most mitochondria abundant tissues in the human body. The kidney is the second organ for mitochondrial abundance since tubular cells require large amounts of energy to maintain their reabsorption function. In both the healthy kidneys and heart, fatty acid oxidation (FAO) and mitochondrial oxidative phosphorylation (OXPHOS) are the preferred pathway to produce ATP, with glycolysis being a minor pathway [10,22]. During acute kidney injury (AKI), tubular cells undergo metabolic reprogramming, as FAO genes are downregulated while glycolytic enzyme gene expression increases to restore the energy supply. However, FAO dysfunction leads to ATP depletion, lipid accumulation, inflammation, and fibrosis [23]. FAO is also modified in endothelial dysfunction [24] and decreased in heart failure; however, in this case, the alternative source of ATP is unclear [25].

Mitochondrial antioxidant defences are crucial for scavenging reactive oxygen species (ROS) generated by the electron transport chain (ETC) and they include enzymatic and non-enzymatic systems [26]. Among the enzymatic systems, superoxidase dismutase (SOD) converts the most dangerous ROS, superoxide anion (•O_2_^−^), to hydrogen peroxide (H_2_O_2_), and H_2_O_2_ is reduced to water by mitochondrial catalase and glutathione peroxidases (a major scavenger in mitochondria to deal with H_2_O_2_ reduction) [27,28]. In this line, physiological low levels of mitochondrial reactive oxygen species (mtROS) can activate survival pathways, whereas excess mtROS cause oxidative stress and eventually cell death, so a balance between mtROS production and scavenging is essential to maintain healthy mitochondria and cells [29,30,31].

## 3. Mitochondrial Biogenesis

Mitochondrial biogenesis is the cellular process by which new mitochondria are produced [32]. However, mitochondria cannot be produced de novo from scratch; they are produced by adding new contents (proteins and membranes) to pre-existing mitochondria, following a fission process similar to prokaryotic binary fission [33]. Mitochondrial biogenesis is a complex process that involves both the mitochondrial and nuclear-encoded proteins and replication of mtDNA, requiring a co-ordinated regulation of both genomes by the transcriptional coactivator peroxisome proliferator-activated receptor gamma coactivator 1-alpha (PGC-1α) [34,35]. PGC-1α expression is altered in kidney and cardiac disease [35,36,37] and, in some cases, exerts its effects through direct interaction/coactivation with PPARs, ERRs, and NRF-1/NRF-2, among other transcription factors [38,39].

### 3.1. Role in Renal Damage

There is considerable evidence that mitochondrion biogenesis is altered in AKI, and this negatively affects kidney function. Healthy proximal tubular cells are rich in mitochondria and express high levels of PGC-1α whose expression is severely reduced in human AKI and in preclinical AKI induced by lipopolysaccharide (LPS), ischemia reperfusion injury (IRI), folic acid overdose, or cisplatin overdose [40,41,42,43,44]. PGC-1α was the transcriptional regulator with the most severely decreased activity in folic acid AKI [44]. In both LPS and folic acid AKI, the reduced PGC-1α expression correlated with the reduced expression of its mitochondrial downstream target genes and negatively correlated with the serum urea levels, suggesting that PGC-1α downregulation during AKI has functional consequences. This was confirmed in PGC-1α knockout mice that showed persistent AKI following saline resuscitation LPS and more severe AKI following folic acid or IRI than wild-type mice [40,41,45]. In this line, the reduced renal mitochondrial mass observed in folic acid AKI was more severe in PGC-1α-deficient mice and negatively correlated with plasma creatinine levels [44]. Additionally, the transgenic overexpression of PGC-1α or treatment with drugs (NAD^+^ precursor Nicotinamide (NAM), AMPK, and Phosphodiesterase inhibitors and anti-TWEAK antibodies) that preserve PGC-1α expression resulted in increased mitochondrial mass and reduced kidney injury in experimental AKI [34,45], suggesting that therapeutic approaches that promote PGC-1α expression and/or mitochondrial mass may be beneficial in AKI.

There is a bidirectional relationship between inflammation and altered mitochondrial biogenesis. Inflammation may directly downregulate PGC-1α, as observed in cultured tubular cells exposed to the inflammatory cytokines TNFα or TWEAK, and in mice injected with TWEAK [40,41]. (TWEAK) is inflammatory cytokines member of the TNF superfamily that signals through its receptor FN14 and is involved in several biological processes, including proliferation, angiogenesis, induction of inflammatory cytokines, and, under some experimental conditions, apoptosis [46,47,48,49]. On the other hand, PGC-1α knockout mice develop spontaneous kidney inflammation as well as more severe kidney inflammatory responses during AKI than wild-type mice [44], supporting the existence of a positive feedback loop between inflammation and PGC-1α downregulation during AKI. Indeed, anti-inflammatory therapies may preserve PGC-1α expression in AKI. Thus, treatment with anti-TWEAK antibodies in folic acid AKI preserved kidney PGC1α levels, reduced kidney inflammation, and improved kidney function [40,50].

**Figure 1:**
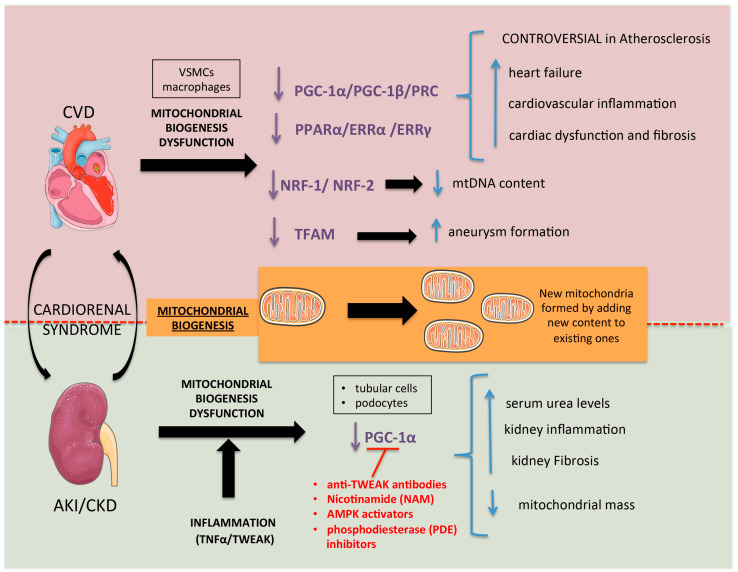
**Therapeutic approaches and key targets in mitochondrial biogenesis in the cardio-renal axis**. Dysfunction of mitochondrial biogenesis transcriptional activators such as the peroxisome proliferator-activated receptor γ-coactivator-1 family (PGC-1α/PGC-1β/PRC) and of transcription factor effectors (PPAR family, ERR family, NRF-1, and TFAM) that also regulate mitochondrial biogenesis may trigger deleterious responses in the kidney and cardiovascular system. (PGC-1α: Peroxisome proliferator-activated receptor-gamma coactivator 1-α/PGC-1β: Peroxisome proliferator-activated receptor-gamma coactivator 1-β/PRC: PGC-1-related coactivator/PPAR: Peroxisome proliferator-activated receptor/ERR: estrogen-related receptors/NRF-1: Nuclear Respiratory Factor 1/TFAM: mitochondrial transcription factor A/TWEAK: Tumor necrosis factor-like weak inducer of apoptosis/NAM: Nicotinamide/AMPK: AMP-activated protein kinase/PDE: phosphodiesterase/TNF-α: Tumor necrosis factor α/VSMCs: Vascular smooth muscle cells/AKI: Acute kidney injury/CKD: Chronic kidney disease).

There is also evidence of altered mitochondrial biogenesis in CKD. Both a metabolomic study in urine from patients with diabetic CKD and a transcriptomic study in microdissected kidney tubule samples from CKD patients of different etiologies showed that mitochondrial biogenesis, FAO genes, and PGC-1α expression were impaired in CKD patients compared to healthy controls [51,52,53]. Furthermore, PGC-1α expression is also reduced in animal models of kidney fibrosis induced by folic acid overdose or by Neurogenic locus notch homolog protein 1 (Notch) overexpression, and tubule-specific overexpression of PGC-1α ameliorated Notch-induced kidney fibrosis [53]. The expression of PGC-1α is also reduced in podocytes of diabetic kidney disease (DKD) patients and in animal models of DKD, but the specific overexpression of PGC-1α in podocytes altered the mitochondrial properties and caused albuminuria and glomerulosclerosis [54]. In addition, a study described the key role of other downstream PGC-1α transcription factors in mitochondrial biogenesis [38,39]. A study in renal cancer cells showed that Mitochondrial pyruvate carrier (MPC) 1 is a novel target gene of PGC-1α. In this study, we demonstrated that the reduced expression of PGC-1α diminished the MPC expression, triggering the impaired mitochondrial respiratory capacity, reducing pyruvate transport into the mitochondrial matrix [55]. In renal cells from TFAM KO mice, we can see the induced cytosolic translocation and activation of the cytosolic cGAS-stimulator of interferon genes (STING) that induced the aberrant packaging of the mitochondrial DNA (mtDNA), cytokine expression, and immune cell recruitment [56]. These findings suggest a critical window of PGC-1α activity for AKI and CKD in regulating renal inflammation and fibrosis (Figure 1).

### 3.2. Role in Cardiovascular Damage

In CVD, mitochondrial biogenesis is decreased as characterized by a reduced mtDNA copy number or reduced metabolic enzymes associated with a decreased ATP production and/or enhanced ROS formation [57]. The main transcriptional activators linked to mitochondrial biogenesis belongs to the peroxisome proliferator-activated receptor γ-coactivator-1 family that includes PGC-1α and PGC-1β and PGC-1-related coactivator (PRC) [58,59]. Overexpression of PGC-1α in mice exacerbated mitochondrial biogenic responses associated with increased nuclear-encoded mitochondrial genes [60]. Germline targeting of PGC-1α and PGC-1β blocked mitochondrial biogenesis in the heart and triggered perinatal lethal heart failure [61].

In atherosclerosis, mitochondrial biogenesis is also modulated [62]. In human atherosclerotic arteries, the gene and protein PGC-1α levels are lower than in healthy subjects [63], especially in symptomatic plaques [64]. In human macrophages, PGC-1α overexpression in response to conjugated linoleic acid (CLA) treatment can block foam cell formation [64]. Vascular smooth muscle cell (VSMC) overexpression of PGC1α in rabbits diminished atherosclerotic lesions, including a decrease in proinflammatory cytokines, adhesion molecules, macrophage infiltration, senescence markers, matrix metalloproteinases (MMPs), and ROS production [65]. However, the role of PGC-1α in atherosclerosis is controversial. In APOE^−/−^ mice, PGC-1α deficiency did not contribute to enhanced atherosclerosis [66]. In PGC-1α^−/−^ApoE^−/−^ mice fed with a high-fat diet, the atheroprotective role of PGC-1α was limited to aged mice [67].

In the proteomic and genomic analysis on Fibulin-4R/R mouse aortas that resemble cutis laxa syndrome (associated with thoracic aortic aneurysm), the mitochondrial protein composition was altered and mitochondrial respiration reduced [68]. Similar results were observed in murine Loeys–Dietz syndrome [68]. Aortas from Fibulin-4R/R mice developed aneurysms and increased ROS production related to a decrease in PGC1α activity [68].

The modulation of PGC-1 coactivators after birth causes mitochondrial morphological derangement and progressive cardiomyopathy [69]. PGC-1α/β deficiency in the adult heart did not result in spontaneous abnormal mitochondrial dynamics or heart failure [69], but accelerated cardiac dysfunction and increased stress after pressure overload after transverse aortic constriction [70]. In contrast, the overexpression of PGC-1α in the vascular endothelial cells of mice induced re-endothelisalization after carotid injury [71]. Moreover, endothelial cells isolated from PGC-1α^−/−^ mice showed an aberrant migration process [71].

PGC-1 coactivators may directly interact with transcription factor effectors such as the PPAR family, ERR family, Nuclear Respiratory Factor 1 (NRF-1), and mitochondrial transcription factor A (TFAM) [72]. PPAR family members have a critical role in mitochondrial biogenesis and FAO in the heart. PPARα deletion and overexpression regulate cardiomyocyte fatty acid metabolism and PPARα deficiency decreases ATP and promotes mitochondria abnormalities and cardiac fibrosis [73,74]. The estrogen-related receptors (ERRα, γ, etc.) are members of the orphan nuclear receptor (NR) family that governs aspects of mitochondrial function by regulating the expression of nuclear-encoded mitochondrial proteins [75]. ERRα knockout mice are susceptible to cardiac dysfunction in response to pressure overload and showed low ATP levels after ischemia [76]. By contrast, ERRγ knockout mice were not viable due to heart failure associated with abnormal oxidative metabolism [77]. NRF-1 and NRF-2 regulate the expression of the ETC complex [78] and activate the transcription of genes related to the replication and transcription of the mitochondrial genome [79]. NRF-1 or NRF-2 deficiency reduced mtDNA content and induced embryonic lethality in mice [80,81]. The cellular myelocytomatosis (c-Myc) transcription factor also controls mitochondrial function and induces NRF-1 and TFAM expression [82]. In mice under pressure overload after an ischemic insult, myocardial c-Myc activation modulated glucose metabolism and mitochondrial biogenesis, decreasing cardiac damage [83]. Mitochondrial biogenesis also modulates aneurysm formation. TFAM is a core mitochondrial transcription factor that has an essential role in mtDNA metabolism [84]. Patients with Marfan syndrome develop aortic aneurysms and have decreased TFAM expression and mtDNA levels in aortas [85]. In fact, in a transcriptomic analysis of aortas from Marfan syndrome (Fbn1c1039g/+) mice, the main pathway highlighted to be related to metabolic function was mitochondrial dysfunction. Moreover, loss of TFAM expression appears to be causative of aneurysm formation, as conditional TFAM-deficient VSMC mice lose their VSMC contractile capacity, develop aortic aneurysm formation, and die prematurely [85]. Restoration of mitochondrial metabolism with the NAD precursor nicotinamide riboside (NR) rapidly reduced aortic aneurysm formation in Marfan syndrome mice [85] and in the AngII-infused model in ApoE^−/−^ mice fed with a western diet [86], increasing the expression of PGC-1α and TFAM. These results suggest a critical role of PGC-1α activity and its coactivators in CVD, however it could be controversial in some cases such as atherosclerotic disease (Figure 1).

## 4. Mitochondrial Dynamics

Mitochondria form a dynamic network in which mitochondria can modulate their morphology to create a tubular network co-ordinated by fission and fusion events to adapt their size, shape, and distribution to changing extracellular and intracellular environments. The balance between fission and fusion events is referred to as “mitochondrial dynamics” [87,88]. Numerous cellular activities, including the cell cycle, apoptotic or regulated necrosis cell death, metabolism, autophagy, and mitochondrial quality control, depend on rapid mitochondrial morphological modifications. The appropriate balance of these processes is essential for maintaining mitochondrial stability and function.

Mitochondrial fusion stimulates the assembly of individual mitochondria that combine their membranes. It takes two steps to fuse the outer and inner mitochondria membranes (IMM). Mitofusin 1 and 2 (MFN1 and MFN2) are located on the outside mitochondrial membrane (OMM) and regulate outer membrane fusion, and protein optic atrophy 1 (OPA1) regulates inner membrane fusion and cristae remodeling, an important determinant of mitochondrial metabolism [89,90].

Mitochondrial fission is the process of mitochondrial division into two separate mitochondrial organelles. Mitochondrial fission 1 (FIS1) and Dynamin-related protein 1 (DRP1) regulate mitochondrial fission. FIS1 is a single-pass transmembrane protein anchored to the outer mitochondrial membrane by its C-terminal region. It binds through MDV1 to DRP1, which is a member of the dynamin superfamily of GTPase-dynamin proteins. DRP1 is located in the cytosol which, during fission, is recruited to the mitochondria, driving membrane constriction in a GTP-dependent manner [91]. Other OMM proteins also participate in the recruitment of DRP1 as the mitochondrial fission factor (MFF), the mitochondrial dynamic proteins of 49 (MiD49) and 51 kDa (MiD51). These last two factors were enough to mediate the fission process in the absence of FIS1 and MFF [92]. Fission facilitates mitophagy, which is the breakdown and recycling of damaged mitochondria. DRP dysfunction may result in the impairment of the fission process, a situation that reduced mitophagy and, finally, induced the accumulation of mtDNA mutations and damaged proteins. In addition, fission-generated fragmented mitochondria produce higher amounts of reactive oxygen species, which may cause oxidative stress and cell injury [93].

Mitochondrial length is associated with the ability to perform OXPHOS. The blockade of mitochondrial fusion through MFN2-depletion boosts mitochondrial fragmentation, reduces mitochondrial membrane potential, lowers oxygen consumption and OXPHOS, and increases dependency on anaerobic glycolysis. Conversely, MFN2 over-expression stimulates mitochondrial metabolism and increases mitochondrial biogenesis and the expression of OXPHOS subunits [94,95]. OPA1 silencing results in similar effects, leading to mitochondrial fragmentation and decreased oxygen consumption via the decreased activity of mitochondrial complexes. At the molecular level, the GTP requirement of the effector proteins OPA1, MFN1, MFN2, and DRP1 may provide a direct link between the cell bioenergetic state and mitochondrial morphology, as most GTPase activities depend indirectly on the overall ATP content [96,97].

### 4.1. Role in Renal Damage

Mitochondrial injury has been linked to renal damage [98]. AKI is accompanied by excessive ROS production, promoting mitochondrial fission protein expression and activation (e.g., FIS1 and DRP1), mitochondrial fragmentation, renal tubular cell injury as well as death [12,98,99,100,101,102]. DRP1 activation and its mitochondrial translocation are an early feature of tubular cell injury, and the suppression of DRP-1 (dominant-negative mutant or siRNA) blocked mitochondrial fragmentation and apoptosis. Importantly, the DRP-1 inhibitor, mdivi-1, reduced mitochondrial fragmentation and protected the kidneys from both ischemia and cisplatin-induced AKI [103]. Mice from the conditional MFN2-deficient-kidney-epithelial-cell mice model have fewer nephrons, although kidney function was generally normal. Nevertheless, the primary proximal tubular MFS2-deficient cells displayed considerable mitochondrial fragmentation and were extremely vulnerable to apoptosis after ATP deprivation [104]. In murine AKI induced by ischemia-reperfusion, emodin was protective by regulating mitochondrial fission through the inhibition of DRP-1 activation [105]. Hence, boosting mitochondrial homeostasis can preserve kidney function. Cisplatin-induced nephrotoxicity is also associated with the increased DRP1-mediated mitochondrial fragmentation, cytochrome c release, and apoptosis of proximal tubular cells [106]. Indeed, the blockade of mitochondrial fission abrogated the cisplatin-induced proximal tubular cell apoptosis and improved kidney function, further supporting the critical role of mitochondrial dynamics in AKI [107].

**Figure 2:**
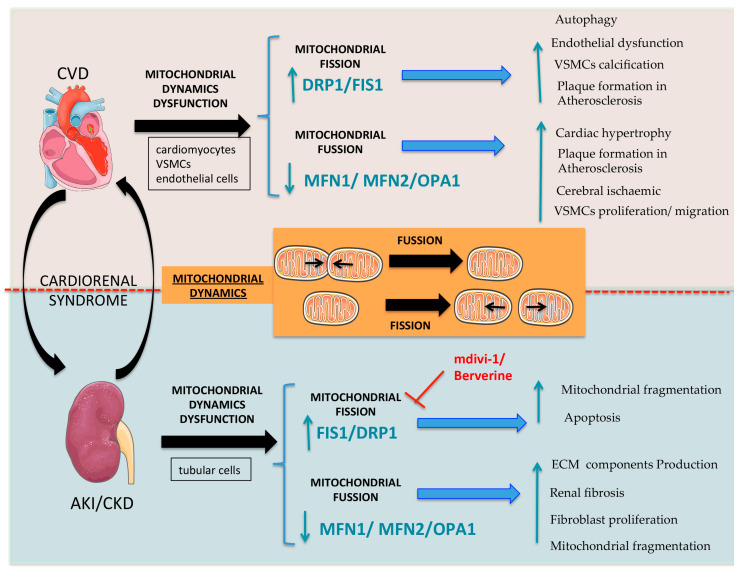
**Therapeutic approaches and key mitochondrial dynamics’ therapeutic targets in the cardio-renal axis.** Modulators of mitochondrial fusion (MFN1/MFN2/OPA1) and fission (DRP1/FIS1) regulate mitochondrial dynamics, and defects on them may trigger deleterious effects in CKD and CVD. (FIS1: Mitochondrial fission 1 protein/DRP1: Dynamin-related protein 1/MFN1: Mitofusin-1/MNF2: Mitofusin-1/OPA1: protein optic atrophy 1/CVD: Cardiovascular disease/AKI: Acute kidney injury/CKD: Chronic kidney disease).

TGFβ1 is a key driver of fibrosis in CKD. In cells with strong TGF1β1 expression, the mitochondrial fusion proteins OPA-1 and MFN2 are frequently downregulated, while the fission protein DRP1 is upregulated [108]. Furthermore, the DRP1 inhibitor mdivi-1 (50 mg/kg twice daily) was reported to reduce renal fibrosis induced by unilateral ureteral obstruction (UUO) for 10 days when administered either prophylactically or therapeutically (i.e., starting 3 days after UUO) [109]. However, despite the reported decreased fibrosis, kidney fibrosis was not quantified. Targeting DRP1 with a pharmacological inhibitor or siRNA increased mitochondrial respiration and reduced the fibroblast activation/proliferation and extracellular matrix production induced by TGFβ1 [109]. By contrast, in another report, Mdivi-1 enhanced the mtROS production induced by hypoxia-stimulated TGFβ1-Smad2/3 signaling in proximal tubular HK-2 cells and was reported to worsen renal fibrosis at day 7 after UUO when used at a dose of 2 mg/kg/day [110]. Overall, the impact of Mdivi-1 on UUO-induced kidney fibrosis remains controversial given the large difference in dose used by different authors and the fact that the report of decreased fibrosis is not supported by quantitative data. In diabetic kidney disease, mitochondrial fragmentation and FIS1 and DRP1 upregulation were reported to contribute to oxidative stress, cellular death, bioenergetic dysfunction, and poor mitochondrial autophagy and biogenesis [111]. FIS1 downregulation improved the mitochondrial morphology and reduced apoptosis in renal cells [111]. Moreover, DRP1 genetic deficiency or treatment with Mdvi1 or Berberine was nephroprotective in diabetic kidney disease [112,113,114]. These findings support the key role of renal mitochondria dynamics (fusion and fission) and associated proteins as therapeutic targets in CKD and AKI (Figure 2).

### 4.2. Role in Cardiovascular Disease

Balanced mitochondrial dynamics are particularly important for maintaining function in cardiovascular cells with high-energy demands, such as cardiomyocytes and VSMCs [115,116]. The role of mitochondrial dynamics during the cardiac development has been extensively studied in genetically modified mice. The systemic knockout of mitochondrial fusion proteins OPA1, MFN1, and MFN2 in mice are embryonically lethal [117,118], and the conditional combined deletion of MFN1/2 in adult cardiomyocytes triggers mitochondrial fragmentation, cardiac hypertrophy, and lethal dilated cardiomyopathy [119,120]. Moreover, gene-trapping MFN2 and OPA1 in mouse embryonic stem cells (ESCs) impaired cardiac development and inhibited cardiomyocyte differentiation [121]. Interestingly, mice with cardiac MFN1 deficiency maintained cardiac function and mitochondrial respiration [122], while cardiac MFN2 ablation resulted in dilated cardiomyopathy and hypertrophy [123,124]. The phenotypical differences between cardiac MFN1 and MFN2 deficiency may result from the role of MFN2 as a Parkin receptor, tethering the endoplasmic reticulum (ER) to mitochondria [125], which is essential for mitochondrial energy metabolism and calcium handling. Opa1delTTAG mutations in a cardiac model of ischemia-reperfusion injury is related to the elevated sensitivity to damage, imbalance in the dynamic mitochondrial Ca^2+^ uptake, and subsequent increase in the Na^+^/Ca^2+^ exchange (NCX) activity [126].

Mitochondrial fission is also critical for cardiac function. Cardiomyocyte-specific DRP1 knockout mice displayed lethal defects in mitochondrial respiration [127,128], likely dependent on overactivation of mitophagy [129] leading to dilated cardiomyopathy [130]. Moreover, mitochondrial fragmentation was observed in the myocardium of patients with heart failure and diabetic cardiomyopathy, in association with an increased expression of Bnip3, DRP1, and MFN1 [131,132].

In atherosclerosis, the expression of Mnf2 was reduced in thrombi from animal models and humans [133,134], while Mnf2 overexpression reduced atherosclerosis in rabbits [133,135]. Mitochondrial damage in VSMCs has been associated with plaque vulnerability in a model of atherogenesis accelerated by CKD [136]. In this sense, the proliferative VSMC phenotype has been associated with decreased MFN2 expression, while the inhibition of mitochondrial fission and of DRP1 reduced VSMC proliferation and migration in an ex vivo aortic ring assay and in rat carotid balloon injury, respectively [137,138]. Interestingly, endothelial dysfunction, a hallmark of atherosclerosis initiation, has also been associated with altered mitochondrial dynamics. Endothelial dysfunctional cells from a diabetic patient displayed the increased expression of Fis1 and fragmented mitochondria [139]. In addition, in a model of diabetic-induced atherosclerosis using ApoE knockout, inhibition of DRP1 reduced endothelial dysfunction, plaque formation [140], and smooth muscle cell calcification [141]. These results suggest that the inhibition of mitochondrial fission or stimulation of mitochondrial fusion may be of therapeutic interest in atherosclerosis.

Altered mitochondrial dynamics was also observed in cerebral ischemia. In a permanent middle cerebral artery occlusion (MCAO) model, MFN2 expression was reduced [142] and mitochondrial fission preceded neuronal loss [143]. Similarly, inhibition of DRP1 improved mitochondrial function, reduced mitochondrial fragmentation, and reduced infarct volume [144,145].

As is the case for mitochondrial biogenesis, there is evidence supporting the involvement of altered mitochondrial dynamics in aneurysm formation. In human abdominal aortic aneurysms, DRP1 expression is higher than in healthy donors [146,147]. Angiotensin II triggers mitochondrial fission in cultured VSMC [138], and this can be prevented by the DRP1 inhibitor Mdivi-1 [138]. In ApoE^−/−^ knockout mice, Mdivi-1 attenuated the DRP1-mediated mitochondrial fission and inflammation in VSMCs and protected from abdominal aneurysms induced by AngII [146]. These results demonstrated that mitochondrial dynamics associated with fission and fusion re ais also critical in several pathological features of the cardiovascular disease (endothelial dysfunction, VSMCs calcification, cell hypertrophy, etc.) (Figure 2).

## 5. Oxidative Stress Originated in Mitochondria

**Figure 3:**
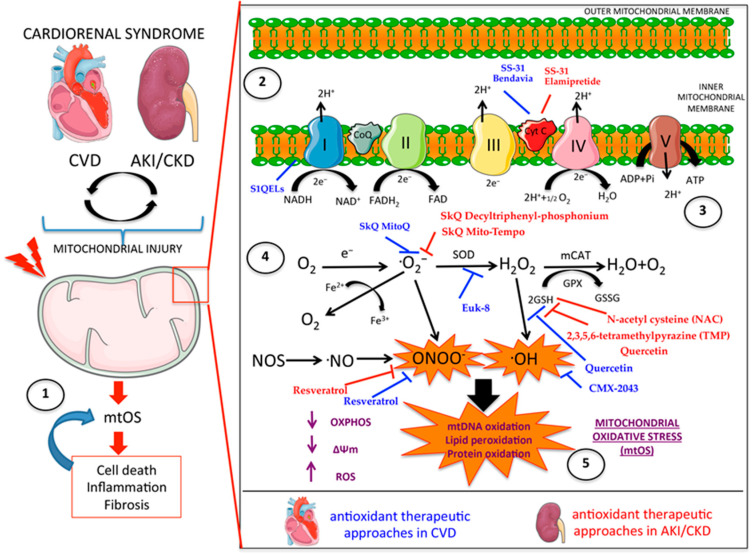
**Oxidative stress is intrinsically linked to the physiopathology of cardio-renal syndrome.** (1) ROS generated by damaged mitochondria induce cell death, inflammation, and fibrosis of renal and cardiovascular tissues. In turn, cellular stress may stimulate ROS production, establishing a vicious cycle between mitochondrial dysfunction and cell injury. (2) ROS production is concentrated in the inner mitochondrial membrane where the ETC is located. (3) Several electrons donated by the coenzymes NADH and FADH_2_ are transferred between the different components of the ETC (from complex I to V) with the transport of protons across the inner membrane, creating the electrochemical gradient that generates ATP. (4) The generation of ROS is characterized by the production of superoxide anion (^•^O_2_^−^) by the transfer of electrons to O_2_. ^•^O_2_ is then converted to hydrogen peroxide (H_2_O_2_) by the enzyme SOD in mitochondria, and H_2_O_2_ is converted to water by GPx or CAT. (5) Excess mitochondrial ROS production can ultimately oxidize mtDNA, proteins, and lipids. Interfering with electron transport by targeting the enzymatic complexes of the ETC and inhibiting cytoplasmic enzymes that lead to excessive free radical production has proven beneficial in limiting the pathological mechanisms triggered by damaged mitochondria. (ETC: electron transport chain/OXPHOS: Oxidative phosphorylation/NADH: nicotinamide adenine dinucleotide/FADH_2_: Flavin Adenine Dinucleotide (FAD) Reduced Form/ATP: Adenosine Tri-Phosphat/SOD: superoxide dismutase/GPx: glutathione peroxidase/ADP: Adenosine diphosphate/mCAT: mitochondrial catalase/ROS: Reactive oxygen species//mt DNA: mitochondrial DNA/mtOS: mitochondrial oxidation/CVD: Cardiovascular disease/AKI: Acute kidney injury/CKD: Chronic kidney disease/ΔΨm: mitochondrial membrane potential).

ROS are produced by mitochondria during the normal functioning of the respiratory chain and OXPHOS, resulting in ATP production. ROS are molecular species with different levels of reactivity between them and need buffering by specific enzymatic systems as well as cellular macromolecules such as proteins and lipids that prevent their accumulation. Physiological ROS regulate pro-oxidative, anti-oxidative, or redox-sensitive signaling pathways including Mitogen-activated protein kinase (MAPK), phosphatidylinositol-3 kinase (PI3K), nuclear factor erythroid 2–related factor 2 (Nrf2), and Restriction Factor 1 (Ref1), but excessive ROS production or deficient scavenging results in oxidative stress and cell damage [148]. Conceptually, dysfunctional mitochondria cause mitochondrial oxidative stress (mtOS); however, oxidative stress may also stress the mitochondria [149] (Figure 3).

### 5.1. Role in Renal Damage

Oxidative stress has been involved in AKI and CKD by favoring a range of pathological mechanisms (cell death, inflammation, and fibrosis) following mitochondrial stress, and several antioxidant agents are kidney protective in preclinical AKI or CKD.

Preventive N-acetyl cysteine administration prevented decreased OXPHOS and FAO, mitochondrial depolarization, pro-oxidative stress, and a GSH deficit to protect mice from folic-acid-induced AKI [150,151]. The specific mitochondrial coenzyme Q10 antioxidants Mitoquinone mesylate (MitoQ) and Mitochondria-targeted carboxy-proxyl (Mito-CP) prevented cisplatin-induced AKI [152]. The natural antioxidant 2,3,5,6-tetramethylpyrazine (TMP) inhibited mitophagy, mitochondrial fragmentation, and oxidative stress and prevented contrast-induced AKI, decreasing tubular cell apoptosis, inflammation, and renal dysfunction [153]. Mitochondrial antioxidants also protected from AKI induced by sepsis, rhabdomyolysis, and ischemia-reperfusion [154,155,156]. The SkQ family of antioxidants, which have mitochondrial specificity and are used at a low dose, protected from ischemia-reperfusion AKI [157]. Mito-Tempo decreased mtROS in cultured tubular cells under oxidative stress and in vivo in ischemia-reperfusion AKI, preventing the decrease in TFAM levels and in the mtDNA copy number, decreasing mitochondrial fragmentation, restoring ATP production, and reducing cytokine release [158]. Mechanistically, TFAM deficiency in renal cells results in the mtDNA release into the cytosol and activation of the Sting pathway, a sentinel innate immune proinflammatory molecule that senses mislocated genomic or mtDNA. Sting promotes kidney damage following mtOS-dependent mtDNA oxidation [56,159,160].

TFAM deficiency is observed in aged murine kidneys [161]. Moreover, aged kidneys show Nrf2 inactivation and elevated lipid peroxidation and 8-OHdG levels resulting from oxidative stress in the context of dysfunctional mitochondria [157,162]. Innovative antioxidant therapies that specifically target mitochondria are being investigated as anti-aging compounds [163,164].

Elamipretide (SS-31) is a water-soluble peptide that accumulates in the inner mitochondrial membrane where it scavenges mtROS independently of mitochondrial membrane potential. SS-31 protects from acute ischemic or obstructive kidney damage. In murine diabetic nephropathy, elamipretide inhibited high-glucose-mediated ROS production, ATP depletion, ∆Ψm decay, and subsequent apoptotic mitochondrial pathway activation, decreasing the oxidative-stress-induced loss of glomerular cells (podocytes and endothelium), mesangial expansion, glomerulosclerosis, and release of renal proinflammatory and profibrotic cytokines [165,166,167]. Interestingly, mitochondrial targeting with Elamipretide protected against kidney ischemic injury in patients with atherosclerotic renal artery stenosis subjected to revascularization with percutaneous transluminal renal angioplasty, resulting in attenuated hypoxia, increased renal blood flow, and improved kidney function [168].

Clinical trials testing the mitochondrial antioxidants Elamipretide, Coenzyme Q10 (CoQ10), and MitoQ in renal diseases have been recently revised [35]. Of the 13 trials, six were completed, two were terminated, three are recruiting, and two have an unknown status [https://clinicaltrials.gov (accessed on 26 April 2023)]. Among them, one studied the role of Elamipretide in cardiac and renal damage in heart failure patients (NCT02914665). In the case of CoQ10, some trials in renal patients described its beneficial role in the oxidative response (NCT00908297), in the incidence of AKI (NCT04445779), and in the improvement of renal function (NCT04972552). MitoQ also has been tested as a dietary supplement for exercise capacity in patients with CKD and heart failure (NCT03960073) and for blood pressure reactivity and vascular function in healthy patients (NCT04334135).

Novel antioxidative nanoparticles (NPs) may also target mitochondria. Several organic (liposome-based or polymeric) and inorganic nanoparticles (QDs, gold nanoclusters (AuNCs), and CeO_2_) have physicochemical properties that may provide sustained and targeted drug delivery systems for dysfunctional mitochondria [169]. As an example, Ceria (CeO_2_) nanoparticles can scavenge ROS by reversible Ce^+3^-Ce^+4^ shuttling. Triphenylphosphonium-conjugated ceria nanoparticles localize into mitochondria and decreased mitochondrial damage in Alzheimer’s disease [170]. Moreover, NPs carrying several compounds such as resveratrol, quercetin, and melatonin scavenge ROS during kidney injury [171,172,173]. These finding demonstrated that the modulation of the mitochondrial oxidative response with several antioxidant compounds, vehicularized or not by NP, have beneficial effects in inflammation, cell death, and fibrosis in renal damage.

### 5.2. Role in Cardiovascular Damage

Mitochondrial DNA damage and oxidative stress have been observed in CVDs [174,175,176,177]. OXPHOS and ATP generation are the main processes that produce ROS in the heart [178]. The blockade of ETC complex I by site I_Q_ electron leak (S1QELs) suppressors inhibit ROS production, protected cardiac function, and decreased infarct size following ischemia-reperfusion [179].

mtROS have a key role in cardiovascular aging. In mice, catalase overexpression (mCAT) in mitochondria reduced cardiac aging [180]. In addition, mice with mCAT overexpression are resistant to angiotensin-II-induced cardiac hypertrophy, fibrosis, and heart failure [181]. In murine metabolic heart disease caused by a high-fat, high-sucrose (HFHS) diet, the transgenic overexpression of catalase in mitochondria prevented contractile dysfunction in the heart [182]. In fact, ROS can decrease the mtDNA copy number by damaging mtDNA replication enzymes [183]. The mtDNA copy number is inversely related to the prevalence/incidence of cardiovascular disease and sudden cardiac death [184,185].

In atherosclerosis, increased ROS production leads to DNA, protein, and lipid oxidation, mainly located in plaque VSMCs [186,187]. mtDNA damage precedes and correlates with atherosclerotic plaque development [188,189]. However, there are controversial opinions about the role of mtROS in mtDNA damage in the context of atherosclerosis progression. Mice with mitochondrial mutations showed no increase in ROS generation despite extensive mtDNA defects [190,191]. In addition, the antioxidant compound MitoQ reduced metabolic syndrome features but had no impact on murine atherosclerosis development [192]. More studies are needed to clarify the role of mtROS in atherosclerosis plaque formation and progression.

A role of mtROS in cardiac disease is better supported by the evidence [175]. In a Chinese population, there was an inverse association between the mtDNA copy number, coronary artery disease, and ROS production [193]. A genomic TFAM blockade caused mitochondrial dysfunction, increased ROS production, and decreased cardiomyocyte proliferation, which were rescued by the mtROS scavenger MitoTEMPO [194]. In cardiac ischemia-reperfusion injury, ROS oxidized thiols in mitochondrial respiratory complex I, inducing an excess ROS production during reperfusion [195]. Mitochondria-specific antioxidant compounds such as 10-(6-Plastoquinonyl) decyltriphenyl-phosphonium (SkQ), Elamipretide, the α-lipoic acid analogue CMX-2043, and MitoQ were protective in cardiac ischemia/reperfusion injury [196,197,198,199,200]. MitoQ also protected endothelial cells from damage induced by chronic exposure to nitroglycerine [201] and controlled blood pressure in hypertensive rats [202]. The SOD/catalase mimetic antioxidant Euk-8 decreased oxidative stress and pressure-overload-induced heart failure in harlequin (Hq) mutant mice [203].

In addition, several lines of evidence indicate that excessive oxidative damage induced by mtROS occurs in atherosclerotic lesions of both animal models and humans [204]. Moreover, mitochondria-targeted anti-oxidants are growing as a new class of compounds for age-related diseases, including atherosclerosis. In this sense, mitochondrial-targeted antioxidant MitoQ ameliorates inflammation, radical oxygen species (ROS) production, and leukocyte–endothelium interactions in atherosclerosis of Type 2 Diabetes (T2D) patients [205]. In addition, mitochondria-targeted esculetin (mito-Esc) antioxidant diminish atherosclerosis in aged ApoE^−/−^ by delaying vascular senescence and pro-inflammatory processes, and by improving mitochondrial biogenesis and function [206]. All these results showed the key role of the mitochondrial oxidative response in the modulation of pathological features of cardiovascular damage including vascular senescence, mtDNA damage, as well as pro-inflammatory processes.

## 6. Mitophagy

Mitophagy promotes mitochondria recycling through the engulfment of whole or fragmented mitochondria by autophagosomes, transferring the mitochondrial cargo to lysosomes for degradation [207,208,209].

**Figure 4:**
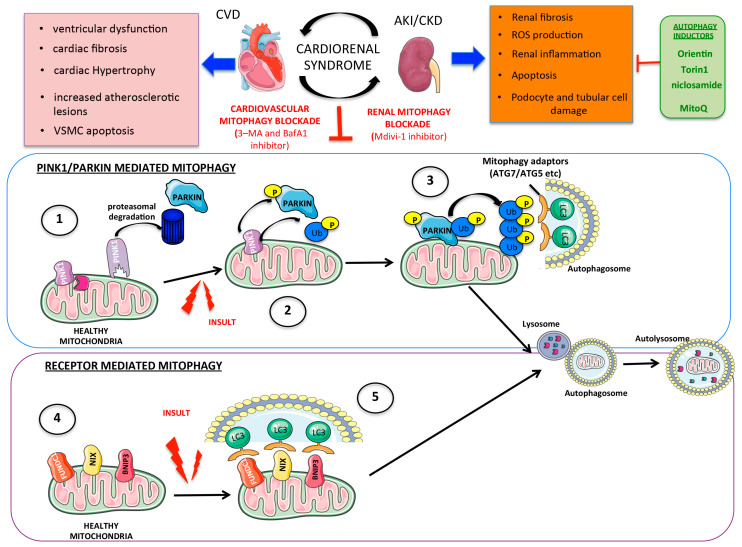
**Mitophagy and cardio-renal syndrome.** (1) Under physiological conditions, PINK1 is cleaved by proteases in the mitochondria. (2) Insults that induce loss of mitochondrial membrane potential trigger PINK1 stabilization and activation in the outer mitochondrial membrane. (3) Activated PINK1 phosphorylates ubiquitin molecules and activates Parkin. Autophagy adaptors subsequently bind to the poly-ubiquitin chains and to LC3 inducing the phagophore formation near mitochondria. (4) and (5) Other mitophagy mediators in the outer mitochondrial membrane include BNIP3, NIX, and FUNDC1 that directly bind to LC3, triggering autophagosome engulfment. Dysfunction of these systems may contribute to CVD and kidney disease and may be targeted therapeutically by autophagy inducers. (PINK1:PTEN Induced Kinase 1/BNIP3: BCL2/adenovirus E1B 19 kDa protein-interacting protein 3/NIX: BNIP3-like protein/FUNDC1: FUN14 domain containing 1/LC3/PARKIN: Parkin RBR E3 Ubiquitin Protein Ligase/3-MA: 3-Methyladenine/BafA1: Bafilomycin A1/ATG5-8: Autophagy-related-5–8/VSMCs: Vascular smooth muscle cells/MitoQ: Mitoquinone mesylate/CVD: Cardiovascular disease/AKI: Acute kidney injury/CKD: Chronic kidney disease).

Most studies investigating the role of mitophagy in physiological or pathological conditions focus on the PTEN Induced Kinase 1 (PINK1)-Parkin RBR E3 Ubiquitin Protein Ligase (Parkin) pathway, which is the most classic mitophagy pathway. PINK1 is a serine kinase that can accumulate in the outer mitochondrial membrane. Upon the loss of mitochondrial membrane potential (ΔΨm), cytosolic Parkin is recruited to the mitochondria by PINK1, resulting in the ubiquitination of mitochondrial proteins, aggregation of p62, and specific binding of microtubule-associated protein 1A/1B-light chain 3 (LC3), thus promoting the recruitment of the autophagosome membrane [210] and initiating the clearance of damaged mitochondria [211]. Mitophagy may also be activated in a PINK-independent manner by mitophagy receptors located in the outer mitochondrial membrane that trigger mitophagy through direct interactions with LC3 on the autophagosome [212]. Nix, BCL2/adenovirus E1B 19 kDa protein-interacting protein 3 (Bnip3), FUN14 domain containing 1 (Fundc1), and others function as mitophagy receptors [213,214,215,216,217,218] (Figure 4).

### 6.1. Role in Renal Damage

In CKD patients and experimental kidney disease, mitophagy-related genes such as PINK1 and Parkin are downregulated [219], and Pink1 and Parkin deletion blocks mitophagy, alters mitochondrial homeostasis, and increases renal fibrosis [220]. In adenine-induced CKD, the myeloid-cell-specific deletion of MFN2 decreased mitophagy, magnified macrophage-derived profibrotic responses, and impaired renal function [220]. In cultured HK-2 cells, Parkin siRNA increased albumin-induced mitochondrial dysfunction and oxidative stress, which were decreased by Parkin overexpression [221]. The single or double Pink1- and Parkin-KO mice display an increased sensitivity to mitochondrial damage, inflammation, and ROS production following kidney ischemia-reperfusion [222]. Murine cisplatin-induced AKI was also more severe in PINK1- and Parkin-deficient mice [223]. Tubular epithelial cells deficient in Pink1/Parkin were more sensitive to cisplatin-induced mitochondrial dysfunction and apoptosis [224]. The deficiency of PINK1/Parkin2 also triggered mitochondrial damage and inflammatory responses [222].

In murine kidney ischemia-reperfusion injury, BNIP3 overexpression induced mitophagy and protected from tubular renal damage [225]. Autophagy-related-5 (ATG5) and autophagy-related-7 (ATG7) are autophagy-related proteins with a key role in autophagic vesicle formation, reducing the degraded macromolecules and organelles which accumulated in the tissue after damage [226]. ATG5 or ATG7 mutant mice displayed deficient mitophagy and developed mitochondrial dysfunction and podocyte and tubular cell vacuolization leading to focal segmental glomerulosclerosis and organ failure [227]. In rats, high-calorie diets decreased mitophagy, caused an abnormal mitochondrial morphology, and increased age-associated kidney injury, while calorie restriction was protective [228].

Altered mitophagy may also contribute to DKD. Optineurin (OPTN) and PINK1 levels were decreased in human diabetic patients; the Mdivi-1 increased renal senescence, while Torin1, an autophagy/mitophagy agonist, was protective [229]. In diabetic mice, Src activation increased the severity of podocyte injury via the suppression of FUNDC1-mediated mitophagy [230]. The antioxidant drug MitoQ upregulated PINK and Parkin expression, preventing tubular injury in diabetic kidney disease (DKD) in vitro and in vivo [231]. The anthelmintic drug niclosamide and its analogues, which are indirect PINK1 activators, exerted renoprotective effects in preclinical type 2 (db/db) and type 1 (streptozotocin) diabetes and also in kidney injury induced by ischemia-reperfusion, UUO, and Adriamycin damage [232,233,234,235,236]. In cultured podocytes, silencing the PINK1/Parkin mitophagy pathway dramatically aggravated mitochondrial ROS and palmitic-acid-induced apoptosis [237]. A bioactive constituent of Fenugreek (Orientin) restored mitochondrial autophagy and reduced high-glucose-induced podocyte apoptosis [238]. Finally, the upregulation of mitophagy components such as PINK1, PARKIN, and related proteins (ATG5 or ATG7) by pharmacological approaches (autophagy agonists, anthelmintic drugs, antioxidants, etc.) restored the damage induced by AKI and CKD (including glomerular damage).

### 6.2. Role in Cardiovascular Damage

Several studies have related autophagic or mitophagy defects with the development of specific cardiovascular disorders [208]. Proteins associated with autophagy, including Beclin1 and different ATG components, were modulated in experimental cardiovascular damage [239]. Heterocygote Becn1^+/−^ mice were more resistant to cardiac damage induced by myocardial infarction than controls [240]. ATG7-overexpressing mice had milder ventricular dysfunction, and cardiac fibrosis and hypertrophy in desmin-related cardiomyopathy [241]. In the hypertensive model of Angiotensin II infusion, Atg5^+/−^ mice exhibited a dramatic cardiac hypertrophy compared to AngII-infused mice [242]. In contrast, the overexpression of other autophagy components could be detrimental. Overexpression of RHEB (a GTP-binding protein that inhibits autophagy by activating mTORC1) in cardiomyocytes increased cardiac injury following myocardial infarction [243].

The PINK1 (PTEN-induced kinase 1)–Parkin (also known as PARK2) signaling pathway is a key receptor-independent pathway that controls mitophagy [244]. PINK1 in mitochondria undergoes rapid proteolytic degradation under normal conditions [72]. In murine cardiac ischemia-reperfusion injury, Pgam5 (PGAM family member 5, mitochondrial serine/threonine protein phosphatase) deletion increased the infarct size [245]. Pgam5 participates in mitophagy. In Park2^−/−^ mice, simvastatin did not activate mitophagy nor protect from ischemia/reperfusion injury [246]. Park2^−^/^−^ mice also showed reduced cardiomyocyte mitophagy and more severe cardiac damage following myocardial infarction [247].

Some protein receptors mediate mitophagy independent of the PINK1–Parkin signaling pathway. Deficiency of the autophagy protein receptor B-cell leukemia/lymphoma 2 protein (BCL2) interacting protein 3 (Bnip3) in mice reduced myocardial damage and preserved the heart structure following cardiac ischemia/reperfusion injury [248]. The deletion of FUNDC1 in mice protected from cardiac ischemia/reperfusion injury through the regulation of platelet activity [249].

In addition, pharmacological inducers of autophagy/mitophagy, such as rapamycin, protected from experimental myocardial infarction [250]. In contrast, autophagy inhibitors as 3–MA and BafA1 exacerbated postinfarction cardiac remodeling and dysfunction [250,251].

There is also a link between mitochondrial dysfunction and atherosclerosis associated with oxidative stress, apoptosis, and deficient ATP synthesis [57]. In atherosclerotic mice (Apoe^−/−^), the endothelial-specific deletion of Atg5 increased endothelial cell apoptosis and atherosclerotic lesions [252]. The overexpression of nuclear receptor subfamily 4 group A member 1 (NR4A1) increased the severity of atherosclerosis through the mitophagy exacerbation that diminished the number of mitochondria and increased endothelial apoptosis [253]. PINK1^−/−^ mice were protected from atherosclerosis and had reduced mitophagy [254]. By contrast, the overexpression of PINK1 or Parkin restored oxLDL-induced VSMC apoptosis in atherosclerosis [57]. The atherosclerotic microenvironment activated rapamycin signaling in macrophages, blocking mitophagy [255]. Other mitophagy protein such as NIX (Bnip3L) that directly interacts with LC3 regulated platelet mitophagy. Nix^−/−^ mice displayed less platelet activation and aggregation and a longer time to complete the thrombotic occlusion of the carotid artery induced by FeCl_3_ [256]. In atherosclerotic aged mice, the mitophagy inducer spermidine reduced mitochondrial dysfunction and atherosclerosis [257]. The blockade of mitophagy induces cardiovascular damage and the overexpression of mitophagy markers improves this injury. However, autophagy upregulation currently is controversial because an excessive activation of this degradative process could have detrimental effects in vascular tissue.

## 7. Conclusions

Mitochondria are dynamic and heterogeneous organelles that are the most important source of oxidative stress and ATP production, and regulate cell function, survival, and death. Mitochondrial dysfunction is involved in the pathophysiology of CKD and CVD. However, a limitation of this review is the paucity of studies that shed light on how mitochondrial dysfunction contributes to the development and progression of the main vascular pathologies that could be affected by kidney injury and vice versa.

Mitochondrial structural/functional abnormalities such as impaired mitochondrial biogenesis, defective mitochondrial dynamics, mitochondrial dysfunction, and oxidative stress contribute to the development and progression of CRS. Several therapeutic approaches using pharmacological strategies to preserve mitochondrial processes successfully slowed the progression of cardiovascular and renal damage in preclinical models. An improved understanding of the molecular links between mitochondrial dysfunction and CVD–CKD interplay may pave the way for clinical studies that test therapeutic interventions aimed at preserving mitochondrial function to protect from CRS.
